# The Shifting Landscape of Natural Health Product Licensing in Canada: The e-Commerce Case of Stem Cell Supplements

**DOI:** 10.2196/86747

**Published:** 2026-09-28

**Authors:** Alessandro R Marcon, Michael Jewer, Timothy Caulfield

**Affiliations:** 1 Faculty of Law, Health Law Institute, University of Alberta, Office 470, Edmonton, AB, T6G 2H5, Canada, 1 780 248 1495

**Keywords:** stem cells, regenerative medicine, Amazon, e-commerce, supplements, natural health products, regulation

## Abstract

Natural health product (NHP) regulations have shifted in Canada, with the aim of reducing “red tape” and enabling market entry. This study assessed how many “stem cell” supplements for sale on Amazon.ca had Health Canada–required natural product number (NPN) licenses compared with vitamin D and “longevity” supplements. It also conducted a marketing overview analysis comparing the Amazon.ca discourse across the 3 product types. Findings show a gap in regulatory licensing compliance between “stem cell” supplements and vitamin D or “longevity” supplements. Marketing discourse also presented potential conflict with regulatory parameters. We discuss the impacts of loosening NHP regulations and the potential for products with weak evidentiary bases to enhance validation through NPN licensing.

## Introduction

Scientifically unsupported commercial activity has long accompanied exciting regenerative medicine research [1]. “Stem cell” supplements are now emerging in online marketplaces, including Amazon. In 2024, our research team assessed “stem cell” supplement marketing on Amazon.com, finding inconsistent adherence to Canadian and American regulations [2]. In Canada, supplements that are natural health products (NHPs) require a licensed good manufacturing practices site and product license (natural product number [NPN]) from Health Canada for marketplace entry [3]. Internal analysis on our 2024 study data found that many “stem cell” supplements lacked traceable NPNs in Health Canada’s NHP database. In 2025, Health Canada implemented NHP regulatory changes to reduce regulatory “red tape” and facilitate market access [4]. This project’s goal was to reassess whether, in July 2026, stem cell supplements on Amazon.ca aligned with NPN license—and broader NHP—regulations.

## Methods

### Overview

In July 2026, we deployed the Apify Amazon Scraper (Apify), a data scraper, to build a dataset of “stem cell supplement” advertisement data for 100 relevant products on Amazon.ca. We also downloaded advertising data from 100 vitamin D and 100 longevity supplements (search: “vitamin D supplement” and “longevity supplement”) to act as control and comparison product types. Vitamin D is popular and typically unrelated to stem cells/regenerative medicine, whereas “longevity” supplements may be associated with aging-related health claims similar to stem cell supplement marketing [2]. Downloaded data, held and analyzed on Google Sheets, included over 400 data columns for each product (including labels, product and use descriptions, and details).

Analysis consisted of describing the central advertising themes across the 3 product types (vitamin D, longevity, and stem cell supplements) and determining whether each product had a legitimate NPN (traceable and active) on Health Canada’s NHP database [5]. To discern central themes, we deployed a straightforward keyword analysis, modeled on our previous research [2], establishing 6 overarching categories: cell activity, health topic, serious health topic, general effect, general qualifier, and science (Table 1). This analysis provided a general marketing overview and potential distinctions between the 3 product types. To identify NPNs, we searched product advertising data, including images (eg, product labels), and, if found, verified NPN legitimacy. If no product NPN was found in the advertisement data, we searched the NHP database for product or brand name(s). Products marketed for animals or exclusively for skin care were excluded, as were duplicates. The data from products with broken Amazon URLs or products listed as “not currently available” were analyzed but excluded from the results.

**Table 1.** Health Canada natural product number (NPN) verification and marketing keywords for 3 supplement types on Amazon.ca.

	Vitamin D (N=78)	Longevity supplements (N=90)	Stem cell supplements (N=80)
Product characteristics			
NPNs found, n (%)	78 (100)	90 (100)	73 (91.3)
Unique NPNs, n (%)	78 (100)	86 (95.6)	70 (87.5)
Unique brands, n	47	44	56
Marketing keywords by category, n (%)			
Cell activity			
Stem cell	0 (0)	2 (2.2)	7 (8.8)
Rejuv_	0 (0)	4 (4.4)	9 (11.3)
Regen_	1 (1.3)	3 (3.3)	6 (7.5)
Nicotinamide^a^	0 (0)	27 (30)	27 (33.8)
DNA	2 (2.6)	8 (8.9)	7 (8.8)
DNA repair	0 (0)	6 (6.7)	5 (6.3)
Cell	10 (12.8)	49 (54.4)	58 (72.5)
Cellular	1 (1.3)	37 (41.1)	38 (47.5)
Health topic			
Aging	2 (2.6)	32 (35.6)	29 (36.3)
Anti-aging	0 (0)	14 (15.6)	17 (21.3)
Immun_	61 (78.2)	30 (33.3)	24 (30)
Inflamm_	2 (2.6)	9 (10)	10 (12.5)
Cardiov_	11 (14.1)	11 (12.2)	10 (12.5)
Stress	6 (7.7)	24 (26.7)	20 (25)
Oxidative	0 (0)	23 (25.6)	23 (28.8)
Energy	11 (14.1)	49 (54.4)	40 (50)
Brain related^b^	7 (9)	29 (32.2)	16 (20)
Mood	6 (7.7)	4 (4.4)	0 (0)
Bone	76 (97.4)	6 (6.7)	6 (7.5)
Joint	7 (9)	8 (8.9)	11 (13.8)
Heart	15 (19.2)	12 (13.3)	8 (10)
Weight loss	0 (0)	1 (1.1)	1 (1.3)
Recover	2 (2.6)	5 (5.6)	5 (6.3)
Longev_	0 (0)	37 (41.1)	12 (15)
Sleep	1 (1.3)	5 (5.6)	3 (3.8)
Gut	1 (1.3)	13 (14.4)	5 (6.3)
Toxi_	0 (0)	5 (5.6)	5 (6.3)
Serious health topic			
Cancer	1 (1.3)	0 (0)	1 (1.3)
Asthma	0 (0)	0 (0)	0 (0)
Depression	0 (0)	0 (0)	0 (0)
Disease	2 (2.6)	2 (2.2)	3 (3.8)
Alzh_	0 (0)	0 (0)	0 (0)
Dementia	0 (0)	0 (0)	0 (0)
General effect			
Heal	73 (93.6)	83 (92.2)	66 (82.5)
Repair	0 (0)	14 (15.6)	18 (22.5)
Cure	1 (1.3)	1 (1.1)	2 (2.5)
Treat	0 (0)	1 (1.1)	3 (3.8)
Prevent	28 (35.9)	3 (3.3)	3 (3.8)
Diagnose	0 (0)	1 (1.1)	0 (0)
Antiox or anti-ox	6 (7.7)	45 (50)	43 (53.8)
Detox	0 (0)	6 (6.7)	6 (7.5)
Cleanse	0 (0)	3 (3.3)	4 (5)
Support	72 (92.3)	84 (93.3)	71 (88.8)
Boost	17 (21.8)	34 (37.8)	33 (41.3)
Strengthen	8 (10.3)	1 (1.1)	3 (3.8)
General qualifier			
Trust	15 (19.2)	16 (17.8)	15 (18.8)
Super	7 (9)	14 (15.6)	13 (16.3)
Good	18 (23.1)	25 (27.8)	14 (17.5)
Pure	28 (35.9)	44 (48.9)	27 (33.8)
Wellness	17 (21.8)	49 (54.4)	25 (31.3)
Natural	45 (57.7)	66 (73.3)	51 (63.8)
Science related			
Science	3 (3.8)	2 (2.2)	11 (13.8)
Scientific	0 (0)	5 (5.6)	8 (10)
Scientist	0 (0)	0 (0)	1 (1.3)
Research	5 (6.4)	15 (16.7)	13 (16.3)
Proven	1 (1.3)	6 (6.7)	6 (7.5)
Evidence	2 (2.6)	1 (1.1)	2 (2.5)
Any science-related^c^	8 (10.3)	20 (22.2)	28 (35)
Tested	21 (26.9)	38 (42.2)	31 (38.8)

a Includes “NAD+,” “NAD,” “NMN,” and “nicotinamide.”

b Includes “brain,” “mental,” “cognit_,” and “memory.”

c Refers to all listed terms excluding “tested.”

### Ethical Considerations

Ethics approval and informed consent were not required as all Amazon ads are publicly visible. No product, brand, or company names appear in this manuscript.

## Results

Nearly all supplements had legitimate NPNs. All 78 vitamin D products and all 90 longevity products had legitimate NPNs. Of the 80 stem cell supplements (56 unique brands), 73 (91%) had NPNs. The 7 (9%) products lacking NPNs came from 3 unique brands and explicitly mentioned “stem cell” in their Amazon.ca marketing. Of these 7 products, 2 were available for purchase but showed a discontinued NPN in Health Canada’s database. An additional 2 stem cell products and 1 longevity product did not produce a legitimate NPN but were excluded for having broken URLs or being unavailable for purchase.

NHP companies selling vitamin D, longevity, and stem cell supplements presented different themes in the advertising discourse (Table 1). Compared to vitamin D products, stem cell and longevity supplements presented greater focus on cell activity and covered a much greater range of health topics (eg, energy, aging, immunity, stress, oxidative, and brain related). Vitamin D products focused almost exclusively on immunity and bone health. Stem cell products presented cell activity with greater frequency than longevity supplements. None of the product types mentioned serious health topics with frequency. The effect descriptors “heal,” “support,” and “boost” were common across all product types, but “cure” and “treat” were scant. The general qualifiers “pure,” “wellness,” and “natural” had a similarly prominent presence across the 3 product types. Stem cell products had the highest presence of science-related discourse (n=28, 35%) compared to longevity (n=20, 22%) and vitamin D (n=8, 10%) (Table 1).

## Discussion

The overwhelming majority of vitamin D, longevity, and stem cell supplements on Amazon.ca in this cross-sectional sample analysis had traceable NPNs in Health Canada’s NHP database. Only stem cell products presented cases of regulatory NPN noncompliance (7/80, 9%). Demonstrating overall regulatory compliance, the marketing for these products scarcely mentioned serious health topics (eg, cancer, dementia) and causal modes of action (eg, cure, treat), which could constitute NHP regulatory breaches [6]. Issues persist, however, about the validation of questionable NHPs by Health Canada’s current regulations and whether obtaining a Health Canada–certified NPN may bolster product credibility.

NHPs certified by Health Canada should not have free rein for deceptive marketing. Both the stem cell and longevity supplements’ marketing in this study frequently mentioned cell activity and a range of health topics. For example, nicotinamide, which is mentioned in approximately 30% of stem cell supplement marketing and 30% of longevity supplement marketing, remains therapeutically speculative despite preclinical development [7]. Vitamin D supplementation also has inconclusive health benefits for nondeficient patients [8]. Canadian NHP regulations restrict marketing that distorts the evidentiary base related to health claims [2]. This project’s broad marketing analysis found science-related discourse in 35% of the stem cell supplement ads and 22% of the longevity supplement ads. Scientific discourse typically works toward promoting evidence around product reliability or efficacy [2]. Notably, stem cell and longevity supplements have a more speculative evidentiary base but more frequently presented appeals to science.

Health Canada’s recent loosening of NHP regulations may be increasing the e-commerce market availability of NHPs, including on Amazon.ca. Correspondingly, the nature of effective surveillance (eg, automated, licensing, risk-based targeting) and enforcement may be fluctuating significantly. The relatively high levels of regulatory compliance shown in this research suggest an opportunity for increasing regulatory pressure around deceptive or overexaggerated product promotion, especially given recent industry-favorable measures to facilitate market access. Ongoing and diligent NHP marketplace surveillance is needed to track the emergence of new products (where compliance may be low) and the corresponding marketing used to promote products with limited, circumstantial, or nonexistent health value for Canadian consumers.

## Abbreviations

NHP: natural health product
NPN: natural product number
